# Hope in Advanced Cancer Patients in the Terminal Phase of Neoplastic Disease and Stability of Basic Mood

**DOI:** 10.3390/jcm9113550

**Published:** 2020-11-04

**Authors:** Bożena Baczewska, Bogusław Block, Beata Kropornicka, Maria Malm, Dagmara Musiał, Marta Makara-Studzińska, Agnieszka Zwolak

**Affiliations:** 1 Chair of Internal Medicine and Department of Internal Medicine in Nursing, Medical University, 20-093 Lublin, Poland; bozena.baczewska@umlub.pl (B.B.); beatak_1966@o2.pl (B.K.); agnieszka.zwolak@umlub.pl (A.Z.); 2 Institute for Family Studies, Faculty of Social Sciences, The Pontifical University of John Paul II, 31-002 Kraków, Poland; bblock@op.pl; 3 Department of Medical Informatics and Statistics with E-learning Lab, Medical University, 20-090 Lublin, Poland; 4 Faculty of Education and Psychology, Maria Curie-Sklodowska University, 20-004 Lublin, Poland; dagmara.musial@poczta.umcs.lublin.pl; 5 Department of Health Psychology, Jagiellonian University Collegium Medicum, 31-501 Kraków, Poland; marta.makara-studzinska@uj.edu.pl

**Keywords:** stability of basic mood, hope, terminal, cancer, patients

## Abstract

The aim of this research is to compare the hope experienced by advanced cancer patients in the terminal phase of neoplastic disease in relation to the stability of their basic mood. The study group consisted of 246 patients, average age 59.5. The youngest respondent was 18 and the oldest was 90. The diagnostic tools used in the work comprised the Personal Card designed by T. Witkowski (PC) and an NCN-36 test (Block’s Hope test), designed by B.L. Block to measure the strength of hope in people struggling with serious life-threatening diseases. The test consists of 4 subscales distinguished by factor analysis. Each subscale consists of 8 items. The test allows an evaluation of hope in the following dimensions: situational dimension (health, thelic-temporal dimension), goals to be achieved in the future, spiritual dimension (spirituality), religious beliefs, and emotional-motivational (affective) dimension (motivations). In cheerful patients who are in the terminal phase of cancer, mood stability does not constitute a major differentiating factor for experiencing hope. In sad people, on the other hand, mood stability affects the intensity of hope—those with an unstable mood are more likely to have a stronger emotional-motivational dimension of hope than sad people with a balanced mood.

## 1. Introduction

The conceptual range of hope is diverse. While some researchers believe that a greater importance in the analysis of hope should be given to the cognitive aspect, others think that the main focus should be on the motivation that leads an individual to take action. For many others, the spiritual dimension of hope is the most important attribute.

In the understanding of Fromm [1], hope is a constant readiness for what is being born, for what is not yet there. It is an intense, unfinished activity, and is accompanied by faith as a belief in the not yet shown possibility of what is not yet available, as a certainty of the uncertain. Hope in this approach is an expression of intense but not yet fulfilled activity, which in a given situation reveals itself at the right time. Hope is also understood as a kind of a goal-oriented thinking in which those who have hope see themselves as capable of achieving a desired goal by working out ways to achieve it by means of specific strategies [2]. Hope is also sometimes referred to as waiting for a dream result or as reacting to an unfavorable outcome of an event that is supposed to take place in the future. It is based on events that have not yet come to an end and refers to something that is supposed to happen in the future and is about to be completed soon [3].

We distinguish three types of affective state: mood, emotions and affections. Mood can be defined as a subjective short or long-lasting emotional state that accompanies existence and gives affective tinge to human activity [4]. Mood is also understood as an affective state characterized by longevity, unintentionality, sprawling character and lack of a clear subjective cause [5]. Watson is the author of a two-factor concept whereby a mood consists of two independent dimensions: positive affect and negative affect [6]. It is worth noting that from this perspective, mood can be understood as a feature and a condition. Mood as a condition is susceptible to the influence of situational factors and is therefore very changeable, while mood as a feature is a relatively permanent disposition and determines which affect generally prevails in an individual’s experience.

An individual’s personal experience is a sum of all experiences and it can embrace being diagnosed with cancer. When analyzing the psychological situation of a person with a diagnosed terminal disease, an analogy can be drawn between experiencing this type of disease and the criteria adopted in the definition of a critical event. Due to the particularly destructive nature of the mutual interaction of elements in the subject’s environment (disturbed relations between the individual and the formerly existing world), optimization of functioning in the reality that is changed by the crisis requires changes that have to be made in order to restore a person’s relative sense of homeostasis. Particularly important in this respect is the spirit of hope in the situational, thelic-temporal and spiritual dimensions [7].

The diagnosis and treatment of cancer pose a serious stress and may lead to changes in the psyche of the patients (including anxiety and depressive reactions) and to the occurrence of various affective states [8,9]. The criticality (traumatism) of the disease considered as a stressful life event is determined by the number, type and duration of changes in the patient’s life. This applies in particular to the terminal phase of cancer.

Hope plays a special role in difficult critical situations. Hence, research on hope is an important international problem and it is attracting a growing number of scientists.

Drawing on the scientific achievements of many researchers and theorists of hope, including Dufault and Martocchio [10,11,12], and referring to the approach proposed by Farrean [13], it can be said that hope, on the one hand, allows a calming down, a build-up of trust, a surrender, and in this way reduces suffering and enables self-protection from unproductive burnout. On the other, it increases faith in the improvement of the situation, gives strength and energy, and mobilizes a call to arms, to self-development and the will to overcome one’s own limitations. At the same time, it can be used to improve the quality of life of seriously ill and dying people, as Ludema [14] evidenced in his dissertation. In the terminal phase of the disease, in addition to the alternating states of resignation and hope typical of the presumed consciousness, there appear states of acute breakdown, depression and existential anxiety.

The main purpose of the research is to compare the hope experienced by advanced cancer patients in the terminal phase of neoplastic disease in relation to stability of basic mood.

The detection of relationships between features of hope and stability of basic mood in people with terminal cancer is of clinical importance. The expectation of a positive relationship between the patient’s general mood and his/her level of hope, especially in the terminal phase of cancer, is particularly important in the experienced existential situation. It seems reasonable that researching hope in people of different basic moods significantly point a direction towards helping them, because it shows to what extent patients need help in the form of psychological or medical intervention. People who are generally always serious or sad will be more exposed to the clinical forms of hopelessness and helplessness and are significantly more at risk of despair. They should be supported quickly, i.e., at the early stage of losing hope, with pharmacological treatment and/or appropriate psychological intervention, as opposed to people with a usually cheerful basic mood. Therefore, learning about the specificity of this relation and of the modifying role of basic mood stability would allow for psychological interventions more tailored to the individual patient’s needs and focused on companionable accompaniment on his/her journey to the other side. Thus, it would enable individualization of therapeutic interactions.

The fear of medical workers about causing despair, the loss of hope and feelings of helplessness, and about aggravating suicidal threat (suicide), are often an excuse that hinders communication with the patient. This fear is sometimes broken by “harsh” communication of the truth about the disease. This has a negative impact on the Doctor-Patient relationship (D-P), which should be a therapeutic relationship. Unfortunately, in extreme cases, it prevents this or causes deep iatrogenic trauma. In a way, the external goal of the proposed study is to counteract improper practices, and to facilitate and strengthen good practices in L-P communication, which translates into enhanced patient quality of life and quality of care in the broad sense.

The specific objectives of the study are: (1) to describe the characteristics of hope experienced by people in the terminal phase of cancer in relation to a balanced basic mood; (2) to provide a comparison of the characteristics of hope experienced by people in the terminal phase of cancer in relation to an unstable basic mood.

## 2. Materials and Method

The research project was approved on 06/2010 by the Bioethics Committee at the Medical University of Lublin (opinion no. KE-0254/225/2010). Its implementation was undertaken by a research team led by Dr Bogusław Block.

The results presented in this article are a part of a larger study and the research project is ongoing. The tests will concern the peri-diagnostic period, oncological treatment, remission and relapse. Longitudinal studies on the dynamics of hope in neoplastic diseases are also currently being conducted.

### 2.1. Material

#### 2.1.1. Selection of Patients for Research

The research was carried out in 2010–2012 among 246 patients in the terminal phase of a neoplastic disease. The terminal phase of the disease is the period of the disease when it is no longer possible of cure and there is an irreversible deterioration of the general condition of the patient with an intensification of physical ailments, combined with reduced mobility. This period is usually about 4–6 weeks.

The subjects were patients in hospice and palliative care units, located in 17 day-care centers and 24-h facilities throughout Poland. The research area was intended to be as wide as possible. These were palliative and hospice care centers located in Poland, but not limited to any region or type of institution. These variables (region, type of institution) were noted in the research, but they are not significant in the framework of the research presented here and in the selection of the analyzed variables. Ultimately, the results of the research were collected from 17 sites out of the 23 of which offered to participate in the research. In those hospice and palliative care units that did not participate in the study, there were no patients in such a psychophysical condition that would have allowed them to be enrolled in the study.

The criterion for selecting people for the study was a previously diagnosed neoplastic disease in the terminal phase. Types of neoplastic diseases have been recorded, but they will be the target of other statistical analyzes. Anyone who consented and was able to logically participate in the interview/study was selected for the research.

The patients’ survival time was determined to be up to about three weeks. Some of the respondents lived longer and others for a shorter time. Among the respondents were people who, after a sudden breakdown in health, died without completing the study.

Before the start of the study, each patient was informed of the study’s anonymity and his or her consent was obtained. Some patients refused to participate in the study. They felt too weak or too tired. In general, however, patients willingly participated in the research. Some patients did not complete the research because their condition worsened from day to day and they were too weak to continue. As stated above, several people died without completing their part in the overall research. Less than 10% of all answers were incomplete due to the patient’s fatigue or non-completion due to a breakdown in health and death.

#### 2.1.2. Scheme of Research

The conducted studies are quantitative, but also refer to the personal experiences of the patient. The reference to the patient’s experiences results from the method of conducting the examination in the form of a “conversation-interview” while noting the test results on the Answer Sheet, and from the introductory instruction referring to the patient’s mood, experience in recent days and his/her personal thoughts. The interview method was as described earlier [15,16]. This interview, apart from diagnostic purposes, also allowed the implementation of therapeutic activities. During the study, a friendly atmosphere prevailed, and honest patient responses were also supported by the ambient conditions. The tests were carried out in rooms where only the researcher and the patient were present. Some subjects, however, had difficulty in understanding the questions and required additional information. The questioned patient did not have to complete the questionnaire personally; the person conducting the research did this, and the time of the meeting was adjusted to the patient’s individual capabilities, ability, and need for support.

When conducting research with these specific people, the main goal of the activity was psychological help and, broadly understood, support for the sick and suffering. The course of the research was more about guiding the patient through his/her experience of survival in the terminal phase of the disease than tiresome questioning. Quite often, the interview turned into a therapeutic conversation with the patient. As a secondary (side) effect, the results were recorded on a standardized battery of questionnaire tests.

The average age of the respondents was 59.5; the youngest was 18 and the oldest was 90. The sociodemographic characteristics are presented in Table 1.

### 2.2. Measures

Methodological triangulation was used to enhance the analysis and the interpretation of findings. Two diagnostic tools were used in the study: Personal Card by T. Witkowski (PC) and the Block’s Hope Test (NCN-36) constructed empirically using the phenomenological method.

#### 2.2.1. Personal Card by T. Witkowski (PC)

As described previously [15,16], Personal Card is a set of data selected from an extensive Personal Questionnaire (Witkowski 1976) and supplemented with new data adjusted to the subject of the new research. The Personal Card for persons in the terminal phase of neoplastic disease contained data describing the group, enabling them to be evaluated as variables for further analysis in terms of their possible relationship with NCN-36 results. The basic goal of the Personal Card was to collect systematized information about the person in the terminal phase of the cancer disease and their environment. The intention was to conduct collective research by way of trained interviewers. It was important that the tool was simple and the information easily accessible.

The Personal Card used in this study consists of 17 questions. In addition to basic information such as age, gender, place of residence, marital status, education, and the time elapsed since becoming ill, variables concerning the emotional-motivational sphere such as basic mood were included. In the range of basic mood, two types of mood were distinguished: a cheerful mood and a sad mood, and within these types cheerful unstable and cheerful balanced, and sad unstable and sad balanced were distinguished.

#### 2.2.2. Block’s Hope Test (NCN-36)

Block’s Hope Test (NCN-36) consists of 36 items. The examined person gives answers on a seven-point Likert Scale. In the test, using factor analysis, 4 subscales were distinguished (8 items in each and 4 buffer questions): (1) health—the situational dimension, (2) goals—thelic-temporal dimension, (3) religious beliefs—spiritual dimension and (4) motivations—the emotional and emotional dimension. Detailed description of the Block’s Hope Test (NCN-36) is presented elsewhere [15,16].

In Block’s Hope Test, the situational dimension is related to uncertain hope for recovery or health improvement, concerns about the effectiveness of the therapy, the level of trust towards doctors, and belief in the effectiveness of medicine (both scientific and nonconventional).

The thelic-temporal dimension deals with hope related to the patients’ attitude towards their future. Such hope comes about because the patients realize that they have many reasons to live, since they are convinced that the future has a lot to offer. They therefore have important goals to achieve in their lives, and they have dreams, plans, and ambitions to fulfill. This component of hope becomes an indirect motivational function, spurs the patients into action and encourages them to put effort into recovery. Through such hope, patients care about their health improvement. They are motivated to cooperate with doctors more efficiently and adhere to their recommendations.

Hope of a spiritual-religious character refers to a Supreme Being or personified God whose presence and benevolence the patients place trust in and lay their hopes in. Patients obtaining high results in this subscale are willing to trust God with their lives and their futures, hence, they subject themselves to God’s love and care. This gives them spiritual support and peace of mind in difficult times. Patients with such a hope look at life from a perspective that goes beyond death, the perspective of eschatological time. Strong spiritual hope may facilitate the acceptance of one’s life and fate, but also at the same time may lead to passivity and apathy.

The affective component of hope makes the patients determined not to give in to anxiety, but rather fills them with courage and endurance so that when they think about the future, they experience inner peace. This component of hope seems to be contrary to feelings of sorrow, depression, and strain resulting from a perceived difficult and uncertain future. The results in the affective dimension point towards the patients experiencing ambivalent states, such as anxiety about an uncertain future, suffering and death, yet also feelings of optimism and courage.

The reliability factor for the test—Cronbach’s alpha—was high: 0.92. In the individual subscales this ranged from 0.72 to 0.86, which indicates the internal consistency of the tool. There is only one approved version of this test. The results ranged from 1 to 7 and interpretations were based on the averaged results. Very strong hope is shown by results between 6.0–7.0, strong 5.0–5.99, moderate 4.0–4.99, vague 3.0–3.99, very vague 2.0–2.99, and lack of hope is in the range between 1.0–1.99. The strength of hope felt by the respondent is reflected in the global result. A patient’s hope profile is created by the results on each of the 4 subscales.

### 2.3. Methods of Statistical Analysis

The governing principle in this regard within this study is that methods of statistical analysis should be selected in the easiest possible way to obtain answers to research questions and verify the hypotheses. It was this recommendation that guided the selection of methods for the analysis of the collected research material.

Statistical analysis of the obtained results was performed with the use of the basic descriptive statistics such as arithmetic mean (M), standard deviation (SD), and quantitative and percentage distributions of the number of results.

Student’s *t*-test was used for statistical analysis of differences. The distribution of the analyzed variables did not differ significantly from the normal distribution. The minimum statistical significance level (*p*) was assumed to be at least 0.05.

## 3. Results

The results of the study of hope experienced by cancer patients in the terminal phase are presented in the following tables: Cheerful balanced and with cheerful unstable mood—Table 2; Sad balanced and with sad unstable mood—Table 3.

As indicated in Table 2, the obtained results differ only slightly in favor of people with a relatively stable mood: the difference is 0.02 to 0.23 points on a 1 to 7 scale. The significance factors of these differences, both on the whole NCN-36 scale and on its subscales, showed no statistical significance. Emotional stability or lability of mood in people who are generally cheerful does not have a significant impact on the strength of hope they experience, even when they find themselves facing a fatal disease with a bad prognosis, such as the terminal stage of cancer.

As shown in Table 3, among people with a sad basic mood, differences in results are bigger and range from 0.01 to 0.35 points in favor of those in a sad unstable mood, with an exception for the subscales G and S. Balanced people have more hope anchored in their religious faith and a little more hope in terms of the goals to be achieved in the future, i.e., in the thelic-temporal dimension. However, these differences showed no statistical significance.

Similar to the overall score, in almost all subscales the results of the differences were not significant. The exception was the F subscale—feelings, i.e., the emotional-motivational dimension (*p* < 0.02), in which the difference in averages is relatively small (0.26). Here the results are more similar, which is evidenced by the low standard deviation (SD = 0.72 and 0.57). This allows the researcher to establish a pattern that sad people with an unstable mood are more likely to have more hope in an emotional and motivational dimension than sad people with a balanced mood.

## 4. Discussion

In the studied group of patients, mood was one of the determinants of hope in people dying of cancer. Cheerful people had more hope than did the sad. Lack of hope and vague hope was manifested only by people in a sad mood [15]. The place of residence of the respondents did not significantly differentiate the strength of hope in people in the terminal stage of neoplastic disease [16]. The aim of the presented research results was to detect the relationship between the scope of hope and the stability of basic mood in people with cancer at the terminal stage of the disease. The analysis of the results suggests that mood stability appeared not to be a significantly differentiating factor in experiencing hope—neither in the group of cheerful people, nor in the group of sad. The only exception is the area of the emotional-motivational dimension of hope, in which sad people with an unstable mood may have more hope than sad people with a balanced mood.

In the literature, more and more papers are appearing on the subject and authors are looking for determinants of the level of hope in patients with cancer. One of them may be the level of pain.

Lin et al. conducted research aimed at the comparison of fitness, mood states and hope levels in Taiwanese patients with and without cancer pain [17]. Patients with cancer who suffered pain reported significantly lower performance levels and higher levels of total mood disorder than did cancer patients who did not feel pain. Among patients with pain, the severity of pain was significantly correlated with the state of physical efficiency and mood, but not with the level of hope. The interference of pain in everyday life was significantly correlated with state of efficiency, mood and level of hope. Pain intensity and pain interference were significantly correlated with every state of mood, and with total mood disorder.

Utne et al. investigated the relationship between mood disorders and pain, hope and quality of life in cancer patients in hospital conditions [18]. The patients were assigned to one of four mood groups. A distinction was made between patients experiencing neither anxiety nor depression, patients experiencing only anxiety, patients experiencing only depression and patients experiencing both anxiety and depression. The researchers evaluated how mood differences are related to pain, hope and quality of life. Accordingly, significant differences in the experience of hope were found between patients with neither anxiety nor depression and those with anxiety and depression.

Fehring et al. conducted research to determine the relationship between spiritual well-being, religiosity, hope, depression and other states of mood in elderly people struggling with cancer [19]. They investigated whether there are differences in states of hope, depression and other states of mood in elderly people with high and low internal religiosity and spiritual well-being. The study saw a consistent positive correlation between internal religiosity, spiritual well-being, hope and other positive moods. There was a negative correlation between internal religiosity, depression and other negative mood states. In elderly patients with a high level of internal religiosity and spiritual well-being there were significantly higher levels of hope and positive moods.

Mickley and Soeken conducted research on religiosity and hope in Hispanic and Anglo-American women with breast cancer [20]. Among Hispanics, neither internal nor external religiosity prevailed in predicting existential well-being or hope. However, internal religiosity was a more important predictor of religious well-being and total spiritual well-being than external religiosity. Among Anglo-American women with breast cancer, internal religiosity was a stronger predictor of well-being and hope.

Herth examined the relationship between the level of hope and coping and other variables in cancer patients [21]. A significant correlation was shown between the level of hope and the level of coping with problems (*p* < 0.05). In addition, the strength of religious beliefs and the performance of family duties were significantly associated with the variables of hope and coping regardless of the environment. The time elapsed since the diagnosis was made and the performance of work duties were not significantly related to the level of hope and to coping with the problems caused by cancer.

Daboui et al. conducted research on mood and hope improvement in women with breast cancer who participated in a group therapy based on studying poetry [22]. During the two-month observation, a statistically significant increase in the hope index and a decrease in stress levels were observed. This study suggests that group therapy based on studying poetry can improve mood and feelings of hope in women with breast cancer. Moreover, the study uncovered the fact that the formation of a small group of patients who uses the mystical motifs of Persian poetry to connect with each other could have lasting positive effects in the long term.

It is important to improve the quality of life during the entire course of a neoplastic disease by adjusting the intervention specifically to the needs of an individual. Targeting interventions may include hope-oriented measures to facilitate positive coping strategies [23].

To sum up, among the determinants of the level of hope in patients with cancer the following aspects should be taken into account: mood and its changeability, the level of pain, internal religiosity, social roles performed and the level of coping with problems. These variables should be considered when building individualized support programs for cancer patients or looking for new areas of influence, by taking advantage of their positive impact on patient condition and on their level of hope.

### Study Limitations

The criterion for selection for research was age 18. The patient had to agree to participate in the study. Some patients refused to participate in the study because they felt too weak. Some patients did not complete the study due to a sudden deterioration in health.

## 5. Conclusions

The following conclusions can be drawn from the conducted research. (1) Emotional stability or changeability of mood in people who were generally cheerful appeared not to be significant for the strength of hope experienced. (2) Sad people with an unstable mood appeared to have significantly stronger hope in the emotional-motivational dimension than did sad people with a balanced mood. (3) On the basis of the conducted research, it can be concluded that the hope of people with a sad but unstable mood can perform a self-regulatory function—it improves mood and thus facilitates coping with suffering, thus compensating for the lack of a positive time perspective in which the desired goals would be located. (4) The field of hope for happy people is characterized by a shift towards hoping to accomplish many small and larger goals before passing on. This feature of hope differentiates both groups of respondents.

An in-depth look at the descriptive part of the analysis of the results, i.e., the fields of hope for happy and sad people with a stable and changeable basic mood, shows that people who do not expect to accomplish many interesting, important or pleasant goals in their future have stronger religious and spiritual hopes. In this field, further qualitative research on the collected material may be of interest. This, however, is beyond the scope of this study.

## 6. Effects on Practice

Assessment of the mental state of patients with cancer should take into account their mood and its stability. This will allow the adjustment psychological support and intervention to the individual needs of the patient. In this way the effectiveness of intervention plans for patients will be improved. Psychological support and intervention can reduce the negative experience of cancer patients and improve their quality of life.

The research results clearly show that the awareness of shortening the perspective of one’s life not so much deprives dying people of hope, but—paradoxically—strengthens it. The results of the research strengthen the argument for telling the truth to the sick person and not hiding it under the pretext that this would destroy the patient’s hope. When asked why this is so, some light can be obtained from a qualitative analysis of the collected research results. This, however, goes beyond the scope of this study. However, it can already be said that informing the patient about the actual state of his/her health in the terminal stage of cancer does not destroy hope but strengthens it. Moreover, this takes on an even more spiritual and religious character, as shown by the results of research carried out in Poland. However, to discern if this regularity also applies to other populations, further research should be carried out. It should be noted that among the respondents, almost all people described themselves as believers—Christians of the Catholic faith. In the light of the conducted research, it turns out that the fear of telling the truth about the terminal stage of the disease to a person with changeable moods because they would despair and lose hope is unjustified; there were no statistically significant differences with regard to the stability of basic mood.

## Figures and Tables

**Table 1:** Characteristics of the studied group.

Socio-Demographic Variables		*n*	%
Age	before 35	14	5.69
	36–50	47	19.11
	51–65	99	40.24
	66–90	86	34.96
Sex	female	150	60.98
	male	96	39.02
Education	elementary	59	24.22
	vocational	59	24.22
	secondary	82	33.33
	BA	17	6.91
	MA	29	11.79
Place of living	rural	54	21.95
	urban	192	78.05
Marital status	single	30	12.20
	married	117	47.56
	widowed	67	27.24
	divorced	32	13.01
Material status	bad	73	29.67
	satisfactory	111	45.12
	good	52	21.14
	very good	10	4.07
Housing	on one’s own	71	28.86
	with family	155	60.01
	other	20	8.13

**Table 2:** Hope in advanced cancer patients in the terminal phase with cheerful balanced and with cheerful unstable mood (N = 106).

NCN-36	Cheerful N = 106				Analysis of Differences			
	Balanced N = 70		Unstable N = 36		Difference M	t	df	*p*
	M	SD	M	SD				
So	4.80	0.66	4.70	0.68	0.10	0.712	104	0.478
H	4.23	1.23	4.00	1.42	0.23	0.833	104	0.406
G	5.16	0.96	5.07	1.05	0.09	0.429	104	0.669
S	5.75	0.96	5.69	0.89	0.06	0.298	104	0.767
F	4.05	0.59	4.03	0.55	0.02	0.189	104	0.857

M—arithmetical mean; SD—standard deviation; t—statistical significance factor; df—degrees of freedom; *p*—statistical significance level. So—overall score in the Block’s Hope Test NCN-36; Subscales NCN–36: H—health, i.e., the situational dimension of hope; G—goals to be achieved in the future, i.e., the thelic-temporal dimension of hope; S—spirituality, religious beliefs, i.e., the spiritual dimension of hope; F—feelings, i.e., the emotional-motivational dimension of hope. The values of each scale ranged from 1 to 7.

**Table 3:** Hope in advanced cancer patients in the terminal phase with sad balanced and with sad unstable mood (N = 140).

NCN-36	Sad N = 140				Difference M	Analysis of Differences		
	Balanced N = 51		Unstable N = 89					
	M	SD	M	SD		t	df	*p* < 0.05
S_O_	4.28	0.79	4.37	0.98	−0.09	0.557	138	0.578
H	3.40	1.41	3.75	1.36	−0.35	1.443	138	0.151
G	4.46	1.12	4.45	1.30	0.01	−0.056	138	0.956
S	5.21	1.15	4.94	1.27	0.27	−1.281	138	0.202
F	4.06	0.72	4.32	0.57	−0.26	2.388	138	0.018

M—arithmetical mean; SD—standard deviation; t—statistical significance factor; df—degrees of freedom; *p*—statistical significance level; S_O_—overall score in the Block’s Hope Test NCN-36; Subscales NCN–36: H—health, i.e., the situational dimension of hope; G—goals to be achieved in the future, i.e., the thelic-temporal dimension of hope; S—spirituality, religious beliefs, i.e., the spiritual dimension of hope; F—feelings, i.e., the emotional-motivational dimension of hope. The values of each scale ranged from 1 to 7.
